# Dietary Protein and Muscle Mass: Translating Science to Application and Health Benefit

**DOI:** 10.3390/nu11051136

**Published:** 2019-05-22

**Authors:** John W. Carbone, Stefan M. Pasiakos

**Affiliations:** 1 School of Health Sciences, Eastern Michigan University, Ypsilanti, MI 48197, USA; 2 Military Nutrition Division, U.S. Army Research Institute of Environmental Medicine (USARIEM), Natick, MA 01760, USA; stefan.m.pasiakos.civ@mail.mil

**Keywords:** hypertrophy, protein balance, musculoskeletal, protein RDA

## Abstract

Adequate consumption of dietary protein is critical for the maintenance of optimal health during normal growth and aging. The current Recommended Dietary Allowance (RDA) for protein is defined as the minimum amount required to prevent lean body mass loss, but is often misrepresented and misinterpreted as a recommended optimal intake. Over the past two decades, the potential muscle-related benefits achieved by consuming higher-protein diets have become increasingly clear. Despite greater awareness of how higher-protein diets might be advantageous for muscle mass, actual dietary patterns, particularly as they pertain to protein, have remained relatively unchanged in American adults. This lack of change may, in part, result from confusion over the purported detrimental effects of higher-protein diets. This manuscript will highlight common perceptions and benefits of dietary protein on muscle mass, address misperceptions related to higher-protein diets, and comment on the translation of academic advances to real-life application and health benefit. Given the vast research evidence supporting the positive effects of dietary protein intake on optimal health, we encourage critical evaluation of current protein intake recommendations and responsible representation and application of the RDA as a minimum protein requirement rather than one determined to optimally meet the needs of the population.

## 1. Introduction

Consuming adequate dietary protein is critical for maintaining optimal health, growth, development, and function throughout life. Dietary protein requirements in healthy adults (≥19 years old) are dictated largely by body mass and lean body mass, as well as net energy balance and physical activity [1]. The Institute of Medicine (IOM) established the current Dietary Reference Intakes (DRIs) for protein in 2005, including the Estimated Average Requirement (EAR), Recommended Dietary Allowance (RDA), and the Acceptable Macronutrient Distribution Range (AMDR) [2]. The EAR for protein is 0.66 g per kg body mass per day (g/kg/d) and is defined as the minimum amount of protein expected to meet the individual indispensable amino acid requirements of 50% of the U.S. adult population. The RDA, however, is 0.8 g/kg/d, and reflects the minimum amount of dietary protein required to meet indispensable amino acid requirements, establish nitrogen balance, and prevent muscle mass loss for nearly the entire (i.e., 97.5%) U.S. adult population [2,3]. The RDA for American adults is similar to international adult protein recommendations established by the World Health Organization (0.83 g/kg/d) [4]. The current protein RDA, however, is often incorrectly applied when used as the definition of recommended intake, rather than its true designation as the required minimum intake. This misapplication is problematic for healthy populations and aging adults, and disadvantageous for those with pathophysiological conditions that would necessitate higher-protein needs. 

Over the past decade, the potential muscle-related benefits achieved by consuming higher-protein diets (i.e., > RDA but within the AMDR) have become increasingly clear. Increased protein intake contributes to greater strength and muscle mass gains when coupled with resistance exercise [5], allows for greater muscle mass preservation when consumed during periods of negative energy balance [6], limits age-related muscle loss [7], and, to a lesser extent, provides a greater muscle protein synthetic response when evenly distributed across meals [5,8]. A prospective, cross-sectional analysis of the National Health and Nutrition Examination Survey (NHANES) database demonstrates inverse associations between animal and plant protein intake and waist circumference, body weight, and body mass index (BMI) [9]. Advances in this field of nutritional science have translated to a greater emphasis on higher-protein diets, protein quality, and supplemental protein in peer-reviewed literature, lay media, and the commercial food market. Despite greater awareness of how higher-protein diets might be advantageous for muscle mass, actual dietary patterns, particularly as they pertain to protein, have remained relatively unchanged in American adults as a whole [10]. The disparity between knowledge and action raises the question of whether this expanded understanding of dietary protein is truly meaningful if scientific data are not translated and ultimately reflected in nutrition guidance and, more importantly, in what people eat. As such, the purpose of this brief communication is to highlight common perceptions and benefits of dietary protein on muscle mass, to address misperceptions related to higher-protein diets, and to comment on the translation of academic advances to real-life application and health benefit. 

## 2. Dietary Protein and Muscle Mass Perceptions

**Figure 1:**
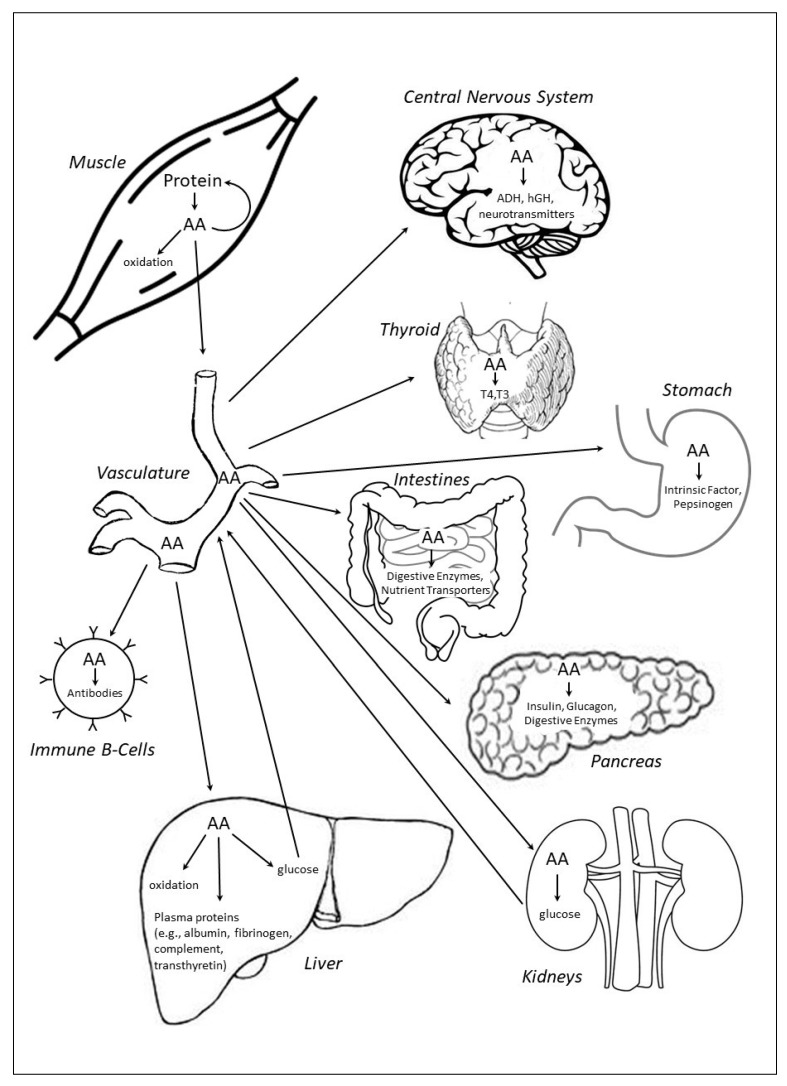
Energy and/or dietary protein restriction induce net muscle catabolism, releasing amino acids for energy production, gluconeogenesis, and synthesis of peptide hormones, plasma proteins, immune system components, and enzymes (representative examples, not an exhaustive list; not drawn to scale). AA, amino acids; ADH, antidiuretic hormone; hGH, human growth hormone; T3, triiodothyronine; T4, thyroxine.

Skeletal muscle protein is dynamic and in constant flux, alternating between states of negative (i.e., muscle protein synthesis < muscle protein breakdown) and positive (i.e., muscle protein synthesis > muscle protein breakdown) protein balance, largely in response to fasting (i.e., postabsorptive) and feeding (i.e., postprandial), respectively. In the postabsorptive state, muscle protein serves as the primary repository of amino acids that is readily catabolized to release free amino acids that can be reincorporated into muscle protein or used to support other critical physiological needs, including serving as an energy substrate through carbon skeleton oxidation, as well as by providing gluconeogenic precursors to support euglycemia (Figure 1). In addition, free amino acids derived from muscle protein breakdown are used in the synthesis of immune system components, plasma proteins, peptide hormones, and intra- and extracellular enzymes. Transient periods of negative protein balance in healthy adults are completely normal and reversed by feeding. The magnitude of the postprandial stimulation of muscle protein synthesis, suppression of muscle (and whole-body) protein breakdown, and shift to a positive protein balance is mediated by dietary protein content, protein quality (i.e., based on an individual protein’s digestibility and absorption kinetics, and abundance of indispensable amino acids), and the format in which protein is consumed (e.g., mixed-macronutrient meal, isolated supplemental intact protein, or free-form amino acids) [6]. The collective optimization of these protein intake-related factors can potentiate the beneficial effects of other protein kinetic stimuli, such as the mechanical and metabolic effects of resistance and aerobic exercise, respectively, resulting in the enhanced remodeling and repair of existing muscle proteins and synthesis of new muscle protein, providing the conditions for muscle maintenance and growth [11].

### 2.1. Current Dietary Protein Recommendations

The current DRIs for protein have been in place since 2005 but are not without limitations. The EAR and RDA were derived from meta-analyses of nitrogen balance studies [12]. The nitrogen balance method has many limitations and tends to overestimate nitrogen intake (via diet) and underestimate nitrogen excretion (via urine, feces, sweat, and integumental loss), thus falsely illustrating nitrogen balance [13]. Nitrogen balance is also considered a crude measure that fails to provide any information as to what occurs within the system to modulate the body nitrogen pool and subsequent balance [14,15]. Likewise, the AMDR for protein (10–35% of total daily energy intake) was established by setting the lower end of the AMDR at the relative amount of protein believed to meet the set RDA of 0.8 g/kg/d, while the upper end is the mathematical difference achieved if carbohydrate (45–65% of energy) and fat (20–35% of energy) are consumed at the lower ends of their respective AMDR (i.e., 100% − 45% − 20% = 35% as the upper end of protein AMDR) [2]. Carbohydrate and fat are important energy substrates and energy balance is critical to optimal health, but this derivation raises uncertainty about the physiological relevance underlying a recommended upper limit for protein consumption at 35% of total energy intake. 

Similarly, the RDA may be sufficient to meet the dietary protein needs of healthy, relatively sedentary young adults, though investigators have argued that this recommendation should be reconsidered based on data from studies demonstrating the inadequacy of the RDA within certain populations when compared to greater requirements derived from the indicator amino acid oxidation method [16]. Accordingly, internationally recognized professional organizations recommend protein intakes on the order of double the current RDA for physically active individuals, including the joint recommendation to consume protein between 1.2–2.0 g/kg/d established by the Academy of Nutrition and Dietetics, Dietitians of Canada, and the American College of Sports Medicine [17]. The International Society for Sports Nutrition also recommends protein intake at levels higher than the RDA for physically active individuals (1.4–2.0 g/kg/d) [1]. The definition of the protein RDA itself draws criticism given that it reflects the minimal amount of protein required to prevent deficiency, rather than an amount which may allow for optimal health. The AMDR does provide for more flexibility in dietary protein intake recommendations in the context of the complete diet, yet most American adults habitually consume protein on the lower end of this range (i.e., 14–16% of total energy intake) [10]. 

### 2.2. Dietary Protein and Physical Activity

The benefits of consuming protein following resistance exercise training have been well-documented, especially as they relate to muscle hypertrophy and function [18]. A recent meta-analysis showed significant positive associations between coupling resistance exercise with post-exercise protein ingestion and total fat-free mass, strength, as measured by one-repetition maximum, and muscle size, as measured by myofiber cross-sectional area [18]. The type and volume of exercise plays a determining role in muscle protein synthetic responses to post-exercise protein ingestion [19,20], as does age [21] and the training experience [18] of the individual. The type of protein consumed also factors into the net anabolic response, given that postprandial muscle protein and whole-body protein kinetic responses to free-form amino acids, isolated intact proteins, and mixed-macronutrient meals all differ [22,23,24]. As reflected in sports nutrition recommendations [1,17], holistic evaluation of varied experimental designs suggests that coupling post-resistance exercise protein ingestion (~20–30 g or 0.25–0.30 g/kg) with habitual protein intakes at ~1.6 g/kg/d promotes favorable muscle adaptations to exercise training [18]. 

### 2.3. Dietary Protein during Energy Deficit

Consuming higher amounts of protein during typical moderate energy-deficient weight loss diets (i.e., 500–750 kcal/d deficit [25]) preserves muscle mass in an otherwise catabolic physiological environment [6]. However, the protective effect of higher-protein diets on muscle and whole-body protein homeostasis is compromised as the severity of energy deficit increases beyond 40% of daily energy needs, as a greater proportion of dietary amino acids are oxidized for energy production, thereby minimizing amino acid availability to support protein balance [26] (Figure 1). However, most adults rarely experience acute or sustained periods of severe energy deficit, except for perhaps acute fasting for religious reasons, poorly-constructed drastic weight loss plans, preparation and/or recovery from bariatric surgery, or scenarios where food availability is severely limited (e.g., victims of natural disasters, emergency responders, etc.). Regardless of the cause, these periods of severe energy deficit usually manifest only for short durations (e.g., 1–3 days) and, therefore, are likely physiologically tolerable. However, if energy expenditures are high and dietary energy and protein intake are limited for extended periods of time, for example during sustained, multi-stressor military operations [27,28], the consequences of severe energy deficit are much more problematic, especially if body mass and fat-free mass loss are so severe that immune system and muscle function and performance are compromised [29,30,31]. During those conditions, prioritizing energy intake, more so than focusing solely on protein per se, is vitally important to help prevent excessive muscle catabolism and conserve muscle function and performance. With moderate energy deficit, however, protein intakes on the order of double the current RDA (i.e., 1.6 g/kg/d) have proved efficacious in preserving muscle mass during weight loss [6].

### 2.4. Pathophysiological Conditions

Inadequate dietary protein intake challenges muscle and whole-body protein balance (i.e., protein synthesis = protein breakdown), negatively impacting muscle mass, function, adaptations to exercise, bone and calcium homeostasis, immune system response, fluid and electrolyte balance, enzyme production and activity, and hormone synthesis. In the absence of sufficient dietary protein intake, muscle is catabolized to provide amino acids to allow for continued endogenous protein synthesis in critical physiological tissues and organs [32] (Figure 1). Certain pathophysiological conditions, such as burns [33], chronic obstructive pulmonary disease (COPD) [34], human immunodeficiency virus/acquired immunodeficiency syndrome (HIV/AIDS) [35], cancer [36], and sepsis [37], also challenge protein homeostasis, albeit the etiology and mechanisms for disrupted protein balance are generally much different from those in healthy adults [38]. Nevertheless, these conditions often induce muscle wasting, suggesting that greater dietary protein intakes may be warranted, with specific recommendations based on the individual patient and clinical scenario. 

While much focus has been placed on adult protein needs in the context of these disease states, the potential benefits of higher-protein intakes extend across the lifespan. Muscle loss and failure-to-thrive are particularly worrisome in the pediatric population, a time typically characterized by rapid growth and development. Recent meta-analysis shows that higher protein intakes in critically ill pediatric patients are associated with positive protein balance, improved clinical outcomes, and lower mortality [39]. These effects manifest at intakes above 1.1 g/kg/d and become more prominent when protein intakes exceed 1.5 g/kg/d. Similarly, unintentional weight loss and decrements in muscle mass in the elderly are predictive of morbidity and mortality, particularly in institutionalized populations [40,41,42]. Provision of dietary protein at or above 1.2 g/kg/d is associated with reductions in unintentional weight loss [43]. Dietary protein supplementation, bringing daily protein intake to 1.5 g/kg, may also be beneficial in mitigating the detrimental body composition changes and muscle mass and function losses associated with sarcopenia [44]. Similarly, consideration should be made for the timing and method of delivery, with isolated, intact proteins providing for a greater anabolic response than mixed meals [45]. While advanced age does limit the postprandial anabolic response typically observed subsequent to protein feeding [46,47], regular intakes above the current protein RDA and consumption of at least 0.4 g/kg (i.e., 0.6 g/kg lean body mass) high quality protein at each meal appear to be critical contributors to preservation of muscle mass and strength that may limit frailty in older populations [42,46,48]. There is also some evidence to support the notion that even higher protein intakes (e.g., 70 g per meal) may be of benefit, in terms of suppressing whole-body proteolysis and enhancing net protein balance [49,50]. 

## 3. Protein Misconceptions and Reality

While the popularity of dietary protein has increased over the past decade or longer, largely because of its role in muscle health, there are still some in the media, clinical practice, and within academia [51] that perpetuate certain risks associated with the protein content of balanced mixed-diets for healthy adults. Common criticisms of greater protein intakes, or with the types of foods dietary protein is derived from, include the potential for detrimental effects of protein on bone, renal function, low-grade inflammation, cardiometabolic disease, and cancer risk. These concerns are generally unfounded with regard to the protein content of the diet and are antithetical to contemporary published data [52,53,54,55,56,57,58,59]. Their persistence, however, and the mislabeling of health detriments to protein itself, as opposed to the whole foods which contribute protein to the overall diet, may underlie lower dietary intakes and thereby contribute to suboptimal muscle integrity. While these associations have been corrected in the scientific literature, other concerns do warrant thoughtful consideration and should be debated in the context of whole foods rather than just ascribing protein foods to labels of “non-dairy animal,” “dairy,” and “plant,” without consideration of the other nutrients these foods provide and their possible links to health and disease [60]. 

Observations of hypercalciuria from nearly a century ago in those consuming greater amounts of meat raised concerns that higher protein intakes resulted in increased bone resorption and, therefore, diminished bone mineral density [61]. It was later hypothesized that greater intakes of sulfur-containing amino acids induce an acidemia that leads to increased bone resorption and calcium release from bone as a compensatory mechanism to buffer reductions in pH secondary to higher protein intake [2]. More recent data suggest that this conclusion is false, as well-controlled studies using stable isotope tracer techniques to assess calcium absorption have shown that the observed hypercalciuria secondary to higher protein intake results from increased calcium bioavailability and greater intestinal calcium absorption potentiated by protein [62,63]. Data from NHANES show that dietary acid load and bone mineral density are not related in adults who consume adequate calcium [64]. In fact, higher protein diets may actually protect against osteoporosis, in part, as a result of the increased hepatic release of insulin-like growth factor 1 (IGF-1) [65]. A recent meta-analysis demonstrated that those with the highest protein intakes had significantly lower hip fracture incidence relative to those with the lowest protein intakes, supporting the assertion that increased dietary protein intake may be protective and serve a critical role in accruing and maintaining bone mineral density [66]. The National Osteoporosis Foundation recognizes the potential benefit of dietary protein on bone, while advocating for continued research, particularly the execution of randomized controlled trials that account for dietary calcium intake [67]. 

Higher-protein diets have also been labeled as damaging to the kidneys. Increased amino acid intake can potentiate renal workload and should be reduced in the presence of established renal disease. However, otherwise healthy kidneys are well-capable of adapting to protein intakes above the RDA and within the AMDR. The kidneys are faced with increased nitrogenous waste as more amino acids are oxidized for energy and/or directed towards gluconeogenesis as the relative percentage of energy intake derived from protein increases. In a recent evaluation of NHANES data, protein intake was directly associated with blood urea nitrogen (BUN) concentrations, but those in the highest decile for protein intake (~1.4 g/kg/d) still exhibited normal BUN (14.8 ± 0.3; reference range, 7–20 mg/dL) [9]. This finding held true across non-dairy animal, animal, and plant protein sources and neither glomerular filtration rate (GFR) nor blood creatinine concentrations were related to dietary protein content. Similarly, a recent meta-analysis of randomized controlled trials with dietary protein interventions showed a small but positive relationship between higher protein intakes (i.e., ≥ 1.5 g/kg/d or ≥ 20% energy intake) and GFR [52]. Overall, current available data suggest higher-protein diets do increase renal workload, but they do not negatively impact kidney health nor increase the risk of developing chronic kidney disease in healthy adults.

Recent studies have raised concerns about the potential for higher-protein intakes to increase systemic inflammation. One large-scale investigation showed that those in the greatest high-sensitivity C-reactive protein (hs-CRP) serum concentration quartile also had higher relative protein intakes than the lowest hs-CRP quartile [68]. However, the mean differences in absolute and energy-adjusted protein intakes between the highest and lowest hs-CRP quartiles were only 1.0 and 1.2 g/d, respectively. Similarly, a large controlled dietary intervention study showed greater reductions in hs-CRP with lower-protein intakes (i.e., 10–15% vs. 23–28% total energy intake), although this protein-based difference was observed only in conjunction with a high-glycemic index diet and not with a low-glycemic index background [69]. In contrast, an analysis of the Framingham Heart Study Offspring Cohort shows an inverse association between dietary protein intake and inflammation and oxidative stress scores, derived from measures of nine inflammatory biomarkers [59]. This potential beneficial effect was observed for higher total and animal protein intakes but was even more pronounced with higher plant protein intakes.

Concern has also been raised regarding potential connections between dietary protein intake and risk of cardiometabolic disease and cancer. These associations are typically confounded by misrepresentation of foods labeled as “protein-rich” which may, by their nature, be overall less-healthful nutrient-sparse food options, providing high amounts of total and saturated fats and processing additives (e.g., nitrates, nitrites, sodium) [60]. To the best of our knowledge, there are no data demonstrating a well-defined association between dietary protein itself and cardiovascular disease [70,71] or type 2 diabetes mellitus [56]. Similarly, methionine restriction (e.g., vegan dietary pattern) may be a viable approach to limit carcinogenic processes and tumor growth [72,73], yet meta-analyses show no link between overall dietary protein intake and incidence of colorectal [57] or breast [58] cancers. Higher protein intakes may, however, exert a protective effect on post-diagnosis survival [74]. A greater emphasis must be placed on dietary protein consumption in the context of overall nutrient-dense, healthy food choices when considering relations to health and disease, as the aforementioned potential connections are influenced heavily by food item quality more so than macronutrient profile [55,75].

## 4. Translation and Application

An assessment of national dietary patterns shows that protein food intakes have remained relatively unchanged over the past decade (i.e., 5.79 ounce equivalents (2005–2006), 5.58 (2007–2008), 5.74 (2009–2010), 5.70 (2011–2012), 5.83 (2013–2014), 5.80 (2015–2016)), as intake data for the latest 2-year cycle are nearly identical to those from 10 years earlier [76,77,78,79,80,81]. This static intake pattern may relate to the regular presentation of recommended protein intakes in a g/d format [82,83], calculated from anthropometrics assumed when the RDA was crafted in 2005 (i.e., 70 kg male, 57 kg female [2]), despite the fact that contemporary measures are significantly different (i.e., 90 kg average male, 77 kg average female [84]). A present-day, moderately physically active, average adult male, consuming protein at the RDA, would have an intake below the low end of the AMDR while maintaining energy balance. In reality, most American adults consume ~14–16% of total energy as protein (1.0–1.5 g/kg/d, depending on age and sex) [10]; an amount greater than the current RDA, but supported as beneficial to muscle and overall health by contemporary research. In fact, the Healthy Vegetarian, Healthy Mediterranean-Style, and Healthy U.S.-Style eating patterns promoted in the 2015 Dietary Guidelines for Americans equate to protein intakes 1.55-, 1.94-, and 1.98-fold greater than the current RDA, respectively (theoretical 19–50 year old female consuming 2000 kcal/d) [85]. If the American adult population, as a whole, consumed protein at approximately 1.6 g/kg/d, as advocated in a recent review from one of the more prominent laboratory groups in this field [5], this would still represent an approximate 17–19% of total energy intake, certainly reasonable based on the current AMDR for protein. Indeed, even increasing to 2.5–3.0 g/kg/d would still fall within the 10–35% of total energy from protein suggested by the AMDR and would provide ample opportunity to optimize muscle health. 

In addition to the dietary protein and skeletal muscle considerations, the protein leverage hypothesis suggests that protein under-consumption increases appetite drive in an effort to ensure sufficient amino acid intake [86]. The unfortunate effect of this response in the absence of increased protein intake is excess energy consumption and resultant positive energy balance. The fact that over 120 million Americans have some type of cardiovascular disease [87], over 29 million are believed to have type 2 diabetes mellitus [88], and approximately 5–7% of the young adult population meets the diagnostic criteria for metabolic syndrome [89] illustrates the need to change how we structure feeding recommendations and encourage compliance with dietary guidance. Improper application of the protein RDA in federal policy, which informs institutional feeding practices, can result in dietary protein intakes that may be sub-optimal for muscle growth and preservation and overall health. As an example, the National School Lunch Act (Section 17(o)(1)) requires that participating programs provide “approximately one-third of the daily recommended dietary allowance” [90]. In practice, this means that American school children are required to be provided with one-third of the minimum amount of dietary protein needed to prevent dysfunction, rather than one-third of the amount which may best support muscle mass, growth, and health. Given the relative cost of protein-rich foods and concerns for cost-efficiency amongst school breakfast and lunch program administrators [91], it is reasonable to expect that protein offerings are reflective of the minimum requirement, rather than robust provision for optimal health. 

Certainly, food choices within macronutrient recommendations are critical, with a needed emphasis on nutrient-dense selections [92]. Similarly, we recognize the traditional association between higher-protein diets and higher meat consumption and the much-needed focus on sustainability and the potential environmental impact of our food sources. With these in mind, we strongly encourage the reevaluation of macronutrient recommendations to best reflect high quality science, basing them on experimental studies over observational data [93]. Implementing reliable macronutrient recommendations for both healthy and diseased populations at all stages of the lifecycle, which engender consumer confidence, can then be followed by greater emphasis on quality food choices within those guidelines. Such action would allow for dietary protein recommendations, and resultant public health policy, best designed to provide for muscle accretion, quality, and preservation throughout the lifespan. A realignment of macronutrient intake recommendations with contemporary research findings would create the foundation for advances in public health.
